# Relevance of Fluorodopa PET Scan in Dopamine Responsive Dystonia and Juvenile Parkinsonism: A Systematic Review

**DOI:** 10.3390/neurolint14040079

**Published:** 2022-12-02

**Authors:** Juan A. Moncayo, Maite Vargas, Juan F. Ortiz, Pablo Granda, Alex S. Aguirre, Jennifer Argudo, Willians Tambo, Gabriela Garofalo, Christian John Capirig, Melisa German-Montenegro, Luis G. Rueda

**Affiliations:** 1 School of Medicine, Pontificia Universidad Católica del Ecuador, Quito 170121, Ecuador; 2 School of Medicine, Colegio de Ciencias de la Salud, Universidad San Francisco de Quito, Quito 170901, Ecuador; 3 Neurology Department, Spectrum Health/Michigan State University, Grand Rapids, MI 49503, USA; 4 School of Medicine, Colegio de Ciencias de la Salud, Universidad de Cuenca, Cuenca 010107, Ecuador; 5 Feinstein Institute, Northwell Health, New York, NY 11030, USA; 6 School of Medicine, Colegio de Ciencias de la Salud, Universidad de Central del Ecuador, Quito 170103, Ecuador; 7 Department of Pharmacology, College of Medicine, Davao Medical School Foundation, Davao City 411002, Philippines; 8 Neurology Department, Augusta University, Medical College of Georgia, Augusta, GA 30909, USA

**Keywords:** dopamine responsive dystonia, segawa syndrome, juvenile parkinsonism, fluorodopa PET scan, FD PET scan, low-income countries

## Abstract

Background: Dopamine Responsive Dystonia (DRD) and Juvenile Parkinsonism (JP) are two diseases commonly presenting with parkinsonian symptoms in young patients. Current clinical guidelines offer a diagnostic approach based on molecular analysis. However, developing countries have limitations in terms of accessibility to these tests. We aimed to assess the utility of imaging equipment, usually more available worldwide, to help diagnose and improve patients’ quality of life with these diseases. Methods: We performed a systematic literature review in English using the preferred reporting items for systematic reviews and meta-analyses (PRISMA) and meta-analysis of observational studies in epidemiology (MOOSE) protocols. We only used human clinical trials about dopamine responsive dystonia and juvenile parkinsonism patients in which a fluorodopa (FD) positron emission tomography (PET) scan was performed to identify its use in these diseases. Results: We included six studies that fulfilled our criteria. We found a clear pattern of decreased uptake in the putamen and caudate nucleus in JP cases. At the same time, the results in DRD were comparable to normal subjects, with only a slightly decreased marker uptake in the previously mentioned regions by the FD PET scan. Conclusions: We found a distinctive pattern for each of these diseases. Identifying these findings with FD PET scans can shorten the delay in making a definitive diagnosis when genetic testing is unavailable, a common scenario in developing countries.

## 1. Introduction

Dopamine responsive dystonia is a group of heterogeneous genetic disorders that present with hyper/hypo kinetic disorders that respond to levodopa. The prevalence of the disease is 0.5–1/1,000,000 person-years; the disease affects females 2–4 times more than men [1]. The clinical features vary depending on the genetic cause of DRD [2]. DRD is a treatable disorder that dramatically responds to LD [3]. A trial dose of 300 mg of LD with a sustained response is key to diagnosing the disorder [3]. Usually, genetic testing is necessary [3].

**Figure 1:**
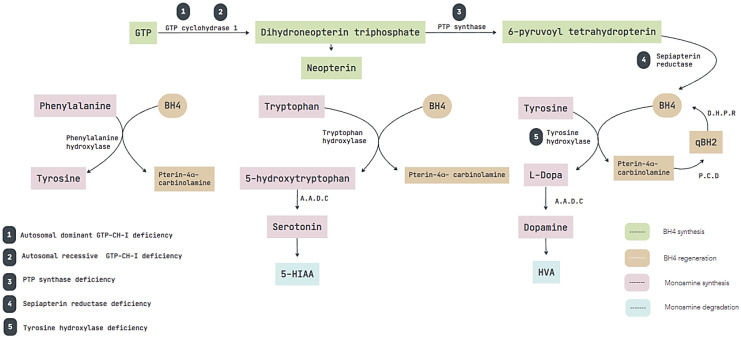
Affected enzymes and phenotypes of DRD. Biosynthesis of BH4 and monoamine neurotransmitters. Abbreviations: AADC, aromatic l-amino acid decarboxylase; BH4, tetrahydrobiopterin; DHPR, dihydropyridine reductase; HVA, homovanillic acid; 5-HIAA, 5-hydroxy indole acetic acid; PCD, pterin-4-α-carbinolamine; PTP, 6-pyruvoyl tetrahydrobiopterin; qBH2, quinonoid dihydrobiopterin.

DRD is divided based on what enzyme is affected, and its severity and response depend on each genotype. There are five variants described so far: (1) Autosomal dominant GTP-CH-1 deficiency, caused by a mutation on the gene GCH-I, a rate-limiting step in the biosynthesis of BH_4_; [4] (2) Autosomal recessive GTP-CH-1 deficiency, this pattern being harsher than the autosomal dominant with an earlier age of onset and more severe clinical presentation characterized by neonatal-onset rigidity, dystonia, truncal hypotonia, spasticity and oculogyric episodes [5]; (3) Tyrosine Hydroxylase deficiency, caused by a loss of function mutation in the Tyrosine Hydroxylase gene, its signs and symptoms are unspecific [6,7]; (4) PTP synthase deficiency, related to a mutation on the PTP gene which affects the second step of BH_4_ synthesis [8]; and (5) Sepiapterin reductase deficiency; this type of DRD is very rare, mainly because it does not always present as such, but instead it usually presents as developmental delay and axial hypotonia, the reason why these patients are diagnosed with cerebral palsy [9,10]. Each enzyme affected is represented in Figure 1.

The term parkinsonism refers to the presence of bradykinesia, dysautonomia, rigidity, and rest tremors [11]. Depending on the physician, population, and methods, the frequency of parkinsonism varies. In the United States population, one study found that the incidence of parkinsonism was 0.8 per 100,000 person-years in the 0–29-year-old age group, and the 30–49-year-old age group was 3.0 per 100,000 person-years [12]. Early-onset parkinsonism (EOPD) is defined as the presence of the signs mentioned above, indistinct from its cause, at age 40 or younger [13]. Juvenile parkinsonism (JP) is defined arbitrarily as parkinsonism signs and symptoms with onset before age 21 years and ‘young-onset’ parkinsonism when its onset is between 21 and 40 years of age [14,15,16,17,18]. JP is an uncommon disorder, usually heterogeneous, and a familial syndrome [14]. There can be many acquired causes of JP, such as exposure to dopamine receptor blocking agents, brain tumors, head trauma, and other secondary causes [11,14,15,16].

The most common subtypes of JP are: (1) Autosomal recessive typical JP, the PARK-parkin, PARK-PINK1, and PARK-DJ1 are the main subtypes, a defective E3 ubiquitin ligase that causes cumulative oxidative damage in tissues with highly mitochondrial activity such as the brain and the heart [19,20,21]; (2) Autosomal dominant JP, caused most commonly by these genetic variants in PARK-SNCA (PARK1, PARK4), PARK-LRRK2 (PARK8) and PARK-VPS35 (PARK17), and are clinically identical than idiopathic PD (iPD) [22]; (3) Other genes involved are the PARK-VPS13C, which is involved in vesicular trafficking and mitochondrial activity [23]. Table 1 describes the most common subtypes of JP.

Undoubtedly, one of the most challenging obstacles that we as doctors in a developing country must face is the lack of resources to offer our patients a timely diagnosis and early treatment. Despite the existence and development of new diagnostic tools for genetic diseases, we must rely on alternative methods that allow us to identify pathologies to treat them most appropriately. Dopamine-responsive dystonia and juvenile parkinsonism are no exception. Although current clinical guidelines present a diagnostic approach based on molecular analysis, the socioeconomic circumstances of developing countries limit us in terms of the possibility of performing these tests. In addition, the poverty that characterizes countless patients does not allow them to access such tools, which adds to the difficulty of medical insurance covering the costs of these innovative tests. Even though it would be ideal to guarantee access to this technology for all patients, we have to work with what we have at our disposal: imaging equipment that, although not the most recommended, allows us to corroborate a diagnosis and improve the quality of life of those who need it.

We focused our attention on the FD PET scan mainly because, even though it is not a very common test to perform, it is more available than genetic testing in developing countries. In addition, this test allows us to have a quick, sensitive, specific marker for a disease that prompts treatment and may go undiagnosed for a significant amount of time if there is no test to confirm the correct diagnosis. Finally, it creates a reliable and measurable finding to compare as the diseases progress.

This systematic review aims to gather information about the differentiation of these two diseases in the setting of a young patient with parkinsonian symptoms and its use in the clinical setting.

## 2. Materials and Methods

### 2.1. Protocol

We carried out a systematic review using the PRISMA and MOOSE protocols. This systematic review was developed and reported in accordance with the Preferred Reporting Items for Systematic Review and Meta-analysis (PRISMA) guidelines which are evidence-based and consist of a minimum set of items focusing on the reporting of reviews evaluating various types of research. Before the formal screening of search results, the protocol for this study was registered in PROSPERO (https://www.crd.york.ac.uk/prospero/display_record.php?ID=CRD42022351259 (accessed on 31 October 2022)) under the registration number CRD42022351259.

### 2.2. Eligibility Criteria and Study Selection

**Figure 2:**
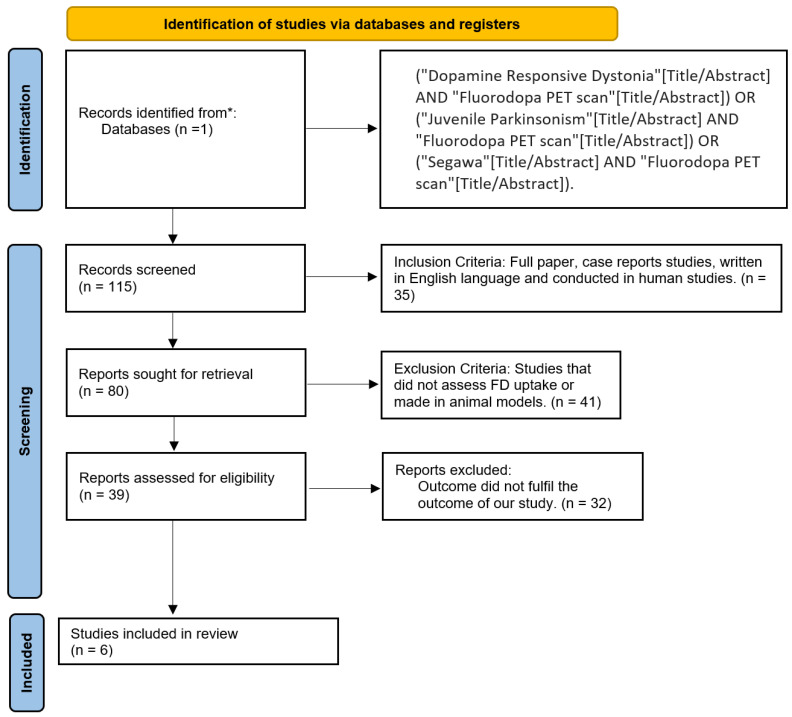
PRISMA flow chart. * Pubmed.

Inclusion criteria were clinical trials conducted on humans and written in English. Exclusion criteria included animal studies, atypical parkinsonism, adult-onset dystonia, and functional neurological disorders, as well as articles that did not fulfill the aim of our study [25,26,27]. As described in Figure 2, we only included articles about dopamine responsive dystonia and juvenile parkinsonism patients in which FD PET scan has been performed. After these filters and discarding 108 studies, six studies bypass our filters.

### 2.3. Database and Search Strategy

We used the PubMed database for this systematic and meta-analysis review. The search was conducted between 1 June and 15 June 2022. We used an advanced search strategy with the following terms: (“Dopamine Responsive Dystonia”[Title/Abstract] AND “Fluorodopa PET scan”[Title/Abstract]) OR (“Juvenile Parkinsonism”[Title/Abstract] AND “Fluorodopa PET scan”[Title/Abstract]) OR (“Segawa”[Title/Abstract] AND “Fluorodopa PET scan”[Title/Abstract]).

### 2.4. Data Extraction and Analysis

We collected the following information from each paper: the author/year, methods, number of participants, and study design. We also extracted the main results, including the outcome measures and limitations of each observational/clinical trial. We analyzed the studies primary and secondary goals and gathered the main conclusions from each study.

### 2.5. Bias Analysis

The assessment of overall risk for bias in this systematic review was conducted with the Newcastle-Ottawa Scale, which divides the risk into three categories: low, moderate, and high; depending on its score, from 0–3, 4–6, and 7–9, respectively. [24] With this tool, the systematic review has a moderate risk of overall bias, demonstrated in Table 2.

## 3. Results

This study performed a detailed analysis of the use of FD PET scans in patients with DRD and JP. After using the PRISMA protocol for systematic reviews and reviewing 115 articles, we selected six case series and excluded 108 studies that fulfilled our exclusion criteria (Figure 2). The risk of bias according to the Ottawa-Newcastle scale is moderate mainly because, by being rare diseases, not much clinical data were available, only case series (Table 2). Takahashi et al., Snow et al., and Sawle et al. described several cases of DRD and EOPD; even though currently this disease is diagnosed in children, these case series are primarily in the adult population probably due to the lack of genetic testing that particular time (1993, 1993, and 1991, respectively). On the other hand, Tanji et al., Hanawaka, and Pal et al. only reported one case each of JP (Table 3).

Takahashi et al. reported three cases from a Portuguese family with DRD, and an asymptomatic carrier of the gene (Table 3). Then, the authors proceeded to measure levels in the cerebrospinal fluid of TH besides the FD PET scan and found that the FD uptake was within the acceptable range compared to healthy control subjects, but tyrosine hydroxylase activity was reduced by 40% in the striatum. [25]

A compilation of case series made by Snow et al., between ten patients with DRD and 18 patients with EOPD, performed FD PET scans and found that the patients with DRD had normal striatal FD uptake compared to EOPD where the structural integrity of the nigrostriatal dopaminergic systems was affected, revealing a decrease in FD uptake (Table 4) [26].

Sawle et al. described six cases of familial DRD plus one idiopathic DRD and found a difference in the uptake of the familial cases where the uptake of FD had a modest decrease in caudate and putamen. As opposed to the idiopathic case, where the uptake was severely decreased and showed a dramatic reduction in tracer activity similar to cases of iPD, this specific case also responded well to LD [30].

On the other hand, in three cases of JP described by Tanji et al., Hanawaka et al., and Pal et al., when comparing the results obtained from FD PET scans of the patients with control subjects, it was observed that the uptake of FD in the putamen was severely reduced; besides this finding, Pal et al. also reported a decreased uptake of the caudate nucleus as described in Table 3. The response to treatment was good, but as expected, due to the structural damage of the dopaminergic neuronal pathway, it was not sustained, and its efficacy decreased over time [28,29,30].

## 4. Discussion

Nowadays, it is well-known that, in DRD, there is a functional defect caused by the lack of enzymes in the dopamine synthesis pathway, as described in Figure 1, preserving the structural integrity of the dopaminergic pathway. On the other hand, JP is characterized by a structural defect of the basal ganglia, confirmed by the difference in uptake of FD, whereas, in DRD, this should be comparable with healthy subjects, compared with JP where the uptake of FD decreased, and thus its clinical response to the administration of LD. This systematic review proposes an alternative to genetic testing focused on low-income countries.

The strength of this systematic review is sustained by an attempt to decrease global-health disparities between low resource countries, and inclusion of usually neglected diseases as it is exemplified by the low quantity of case reports found. A call to report and study these underdiagnosed entities is warranted. The weaknesses of this paper can be found in the limitations described and bias assessment tool used.

The limitations of this systematic review were that we used case series, increasing the chance of bias, and no clinical trials were found. Another strong limitation was that the cases of JP were less than DRD; thus, in order to compensate for this discrepancy, we also used EOPD cases.

## 5. Conclusions

There is a clear pattern of decreased uptake in the putamen and caudate nucleus when using the FD PET scan in cases of JP, compared with DRD where the uptake of this marker is slightly decreased but comparable to normal subjects. The availability of FD PET scans can shorten the period for these exclusion diseases to come to a definitive diagnosis. In real world clinical setting, especially in these rare diseases, the knowledge of giving a name to the pathology can ease the uncertainty felt by the patient and family. When genetic testing is not readily accessible, this can become a valuable tool to help the diagnostic algorithm or for academic purposes. Low-income countries, like Ecuador, could benefit from this approach.

## Figures and Tables

**Table 1:** Subtypes of typical Juvenile Parkinsonism.

Typical Juvenile Parkinsonism	Subtype	Clinical Symptoms
Autosomal Dominant	PARK SNCA (alpha synuclein)	These three subtypes are clinically indistinguishable from idiopathic Parkinson Disease. However, patients with the PARK-SNCA may have a higher rate of non-motor symptoms, atypical signs, and cognitive decline compared to the other two [24].
	PARK-LRRK2 (Leucine Rich Repeat Kinase 2)	
	PARK VPS35 (Vacuolar protein sorting-associated protein 35)	
Autosomal Recessive	PARK-parkin (Parkin—E3 ubiquitin ligase)	These three subtypes are clinically indistinguishable between them.The most common reported symptoms are bradykinesia, tremor, rigidity, dystonia, and postural instability [24].Non-motor symptoms usually are more frequently reported in PARK-DJ1 (57%) and PARK-PINK1 (42%), compared to PARK-parkin (13%) [24].
	PARK-PINK1 (PTEN-induced putative kinase 1)	
	PARK-DJ1 (DJ1 oncogene)	

**Table 2:** Newcastle-Ottawa Scale.

Study	Newcastle-Ottawa Scale			Overall Risk of Bias
	Selection (Total 4)	Comparability (Total 2)	Outcome/Exposure (Total 3)	(Total Score)
Takahashi et al., 1993, USA. [28]	***	-	***	Moderate (6)
Snow et al., 1993, USA. [29]	***	*	****	Low (8)
Sawle et al., 1991, USA. [30]	**	**	****	Low (8)
Tanji et al., 1998, Japan. [31]	*	*	***	Moderate (5)
Hanawaka et al., 1996, Japan. [32]	-	*	**	High (3)
Pal et al., 2002, USA. [33]	*	*	****	Moderate (6)

Newcastle-Ottawa Scale. Score: 0–3: High Risk for Bias, 4–6: Moderate Risk for Bias, 7–9: Low Risk for Bias. ** Good Quality: 3 or 4 stars in selection domain AND 1 or 2 stars in comparability domain AND 3 or 4 stars in outcome/exposure domain, Fair Quality: 2 stars in selection domain AND 1 or 2 stars in comparability domain AND 2 or 3 stars in outcome/exposure domain, Poor Quality: 0 or 1 stars in selection domain OR 0 stars in comparability domain OR 0 or 1 in outcome/exposure domain.

**Table 3:** Description and age when scanned of the Case Series.

Author, Year, Country	Number of Cases Described	Family History	Age When Scanned
**Takahashi et al., 1993, USA.** [28]	**DRD** 3	Yes	16 13 71
**Snow et al., 1993, USA.** [29]	**DRD**: 10 **EOPD**: 18	**DRD**: Yes Yes No Yes Yes Yes No Yes Yes Yes **EOPD**: Yes No Yes No No No No No No No No No No No No No No No	**DRD**: 43 34 42 17 37 41 47 15 63 63 **EOPD**: 27 27 21 53 45 35 36 46 38 33 34 36 45 41 43 43 39 47
**Sawle et al., 1991, USA.** [30]	**DRD**: 7	No Yes Yes Yes Yes Yes Yes	18 47 66 58 25 19 19
**Tanji et al., 1998, Japan.** [31]	**JP**: 1	N/A	17
**Hanawaka et al., 1996, Japan.** [32]	**JP**: 1	Yes	27
**Pal et al., 2002, USA.** [33]	**JP**: 1	N/A	14

DRD: Dopamine Responsive Dystonia, JP: Juvenile Parkinsonism, EOPD: Early-Onset Parkinson’s Disease.

**Table 4:** Treatment, differences between FD PET Scans, and genetic testing of the case series.

Author, Year, Country	LD Response *	FD PET Scan Uptake	Genetic Testing
**Takahashi et al., 1993, USA.** [28]	**DRD:** +++ +++ Not treated	**DRD:** FD uptake was within the acceptable range compared to healthy control subjects, but tyrosine hydroxylase activity was reduced by 40% in the striatum.	Not described
**Snow et al., 1993, USA.** [29]	**DRD:** ++ +++ +++ +++ +++ +++ +++ N/A N/A N/A	**DRD:** Normal striatal FD uptake compared to EOPD where the structural integrity of the nigrostriatal dopaminergic systems was affected, revealing a decrease in FD uptake.	Not described
**Sawle et al., 1991, USA.** [30]	**DRD:** ++ ++ ++ ++ ++ ++ ++	**DRD:** Difference in the uptake of the familial cases where the uptake of FD was modest and decreased uptake in caudate and putamen as opposed to the idiopathic case where the uptake was severely decreased and showed a dramatic reduction in tracer activity than in JP.	Not described
**Tanji et al., 1998, Japan.** [31]	**JP:** +++	**JP:** FD accumulation and its uptake rate constant into the putamen were markedly decreased bilateral. FD uptake was preserved only in the bilateral lower parts of the caudate nucleus.	Not described
**Hanawaka et al., 1996, Japan.** [32]	**JP:** ++	**JP:** A marked decrease in uptake and metabolites was observed, especially in the striatum at the level of the putamen.	Not described
**Pal et al., 2002, USA.** [33]	**JP:** +++	**JP:** Severe decrease in FD uptake in the caudate nucleus and in the putamen. FD uptake in the caudate had decreased by almost 50% of values considered normal, while uptake in the putamen was only 28% of that expected in a normal subject.	The parkin gene was screened extensively. Sequencing of the 12 exons from genomic DNA did not show any mutations.

FD: Fluorodopa, DRD: Dopamine Responsive Dystonia, JP: Juvenile Parkinsonism, EOPD: Early-Onset Parkinson’s Disease, LD: Levodopa, PET: Positron Emission Tomography. * Levodopa Response: ++ = Moderate. +++ = Good.

## Data Availability

Not applicable.

## Abbreviations

FD	Fluorodopa
DRD	Dopamine Responsive Dystonia
PET	Positron Emission Tomography
PD	Parkinson’s Disease
JP	Juvenile Parkinsonism
LD	Levodopa
TDH	Tyrosine Hydroxylase Deficiency
TD	Tyrosine Hydroxylase
SNpc	Substantia Nigra Pars Compacta
EOPD	Early-Onset Parkinson’s Disease
iPD	Idiopathic Parkinson’s Disease
GTP-CH-I	Guanosine Triphosphate-Cyclohydrolase 1
PTP	Pyruvoyltetrahydropterin
PRISMA	Preferred reporting items for systematic reviews and meta-analyses
MOOSE	Meta-analysis of observational studies in epidemiology
